# Looking into the black box of “Medical Innovation”: rising health expenditures by illness type

**DOI:** 10.1007/s10198-022-01447-9

**Published:** 2022-03-17

**Authors:** Friedrich Breyer, Normann Lorenz, Gerald J. Pruckner, Thomas Schober

**Affiliations:** 1 Department of Economics, University of Konstanz, P.O. Box 135, 78457 Konstanz, Germany; 2 University of Trier, Trier, Germany; 3 Johannes Kepler University of Linz, Linz, Austria; 4 Christian Doppler Laboratory for Aging, Health, and the Labor Market, Linz, Austria; 5 Auckland University of Technology, Auckland, New Zealand

**Keywords:** Health care expenditures, Medical innovation, Cost of dying, H51, J11, I19

## Abstract

There is agreement among health economists that on the whole medical innovation causes health care expenditures (HCE) to rise. This paper analyzes for which diagnoses HCE per patient have grown significantly faster than average HCE. We distinguish decedents (patients in their last 4 years of life) from survivors and use a unique dataset comprising detailed HCE of all members of a regional health insurance fund in Upper Austria for the period 2005–2018. Our results indicate that among decedents in particular, the expenditures for treatment of neoplasms have exceeded the general trend in HCE. This confirms that medical innovation for this group of diseases has been particularly strong over the last 15 years. For survivors, we find a noticeable growth in cases and cost per case for pregnancies and childbirth, and also for treatment of mental and behavioral disorders. We discuss whether these findings contradict the widespread interpretation of cost-increasing innovations as “medical progress” and offer some policy recommendations.

**Supplementary Information:**

The online version contains supplementary material available at 10.1007/s10198-022-01447-9.

## Introduction

Health care expenditures (HCE) have been rising considerably faster than GDP in most OECD countries over the past 40–50 years ([5], Table 1.2). In particular, in countries with a large share of public expenditures such as the German speaking ones (Austria, Germany, and Switzerland), the gap between the average annual growth rate of per-capita HCE and the respective growth rate of GDP was near the top of the OECD countries with values between 1.7 and 2.7% over the period 1970–2008. Accordingly, the share of HCE in GDP has increased enormously in all these countries and now amounts to between 10.4 and 12.4%.^1^ Extrapolating this trend into the coming decades suggests that health care financing might become a controversial political issue in the near future.

While it is still debated among health economists to what extent population aging contributes to the growth in HCE (for the opposite views, see, e.g., Zweifel et al. [23] and Breyer et al. [2]), there is much more agreement on the fact that medical innovation is a factor that causes HCE to rise. However, as Chernew and Newhouse [5] argue in their comprehensive survey, technology (and thus the rate of innovation as such) is hard to measure, so that there are essentially two approaches to demonstrate the role of medical innovation in explaining rising HCE: in the *residual approach*, calendar time is used as a regressor in the expenditure equation, and the estimated coefficient for the trend variable is interpreted as the contribution of medical technology to the overall increase in HCE. In contrast, papers using the *affirmative approach* look at how the treatment of specific diseases such as heart attacks has evolved over time and how this has affected the costs of treating the respective patients.

Neither of these approaches is fully satisfactory in measuring the contribution of medical innovation to the growth in HCE: in the residual approach, time is used merely as a proxy, but this variable will pick up the effects on HCE of all other variables which develop over time and are not explicitly included in the regression. Thus, it does not answer the question to what extent medical innovation raises expenditures and thus gives no hint at possible interventions which could be suitable to slow down the expenditure trend. On the other hand, when the focus is only on a few specific diseases, it is not clear how the results emanating from this research can be generalized to HCE in total.

The present paper adopts an intermediate approach. We proceed from research by Lorenz, Ihle, and Breyer [18], who use a panel dataset from a large German sickness fund and find that age-specific HCE have increased particularly strongly in certain age groups, especially the 60 to 80 years old, when they are in their last 3–4 years of life. HCE towards the end of life have been a topic of health economic research ever, since Ginzberg’s [13] outcry about the high cost of dying.^2^

In fact, medical expenditures peak in the last years of life: Although in most developed countries, little more than 1% of the population dies in any given year, this group typically accounts for about 10% of total HCE. If the analysis is extended to all people in their last 4 years of life, the expenditure share increases to about 20% of the total (see, e.g., the papers in French and Kelly [9] and French et al. [10]).

Focusing on the relatively small share of the population (people near their death), which accounts for an over-proportional share of HCE seems to be a promising approach for opening the black box of “medical innovation” and attacking the question for what types of patients treatment costs have risen so much that this has contributed appreciably to the overall HCE growth. Moreover and more importantly, for patients who were about to die, it is easier to identify one particular illness with which their HCE can be associated, namely the ICD code specified as either the cause of death or, alternatively, the primary disease treated during their last hospital stay.^3^ Thus, the research questions to be answered in this paper are:

1. What were the most frequent causes of death and how have HCE for the respective patient groups evolved over time?
2. How has the number of patients who died from any of these diseases evolved over time?
3. For those diseases with the highest expenditure growth, are these high growth rates primarily caused by certain age groups?

The similar procedure for patients who survived a given hospital stay for more than 4 years is more difficult: for these patients, the error committed by attributing total HCE in the respective year to the (main) illness which was treated during one particular hospital episode is probably larger. However, given that the bulk of HCE is caused by this group of patients, we perform the analysis for this group, too.

Our analysis is inherently descriptive. We do not attempt to explain either individual or societal HCE, an endeavor which would require searching for “causal” factors such as income, education, social class, or even the determinants of illness. Instead, the purpose of this study is to break down the trend in total HCE over time to the development in groups of patients suffering from different, but widespread diseases.

We draw on a unique data set from a large regional health insurance fund in Austria, more precisely the province (“Bundesland”) of Upper Austria. The Upper Austrian Regional Health Insurance Fund (OOEGKK), which covers about 75% of the regional population (roughly 1 million insured persons in any given year), provides detailed data on individual health care utilization in the inpatient and outpatient sector for the years 2005–2018.

The remainder of this paper is organized as follows. In “Literature”, we give a short survey of the existing literature on the relationship between medical innovation and HCE. In “Research design”, we describe the data and explain the empirical strategy of estimating the determinants of HCE. The regression results are presented in “Results”, and in “Discussion, limitations, and conclusions”, we discuss the results and conclude.

## Literature

As mentioned above, Chernew and Newhouse [5] distinguished between the residual approach and the affirmative approach to estimate the effect of medical innovation on the overall increase in HCE. The former approach goes back to the seminal paper by Newhouse [19], who tried to explain the 780% growth of per-capita HCE in the U.S. over the period 1940–1990, and found that only about one-quarter could be explained by an increase in income and even including other determinants such as population aging and the spread of health insurance could explain less than half of the expenditure growth. Newhouse further noted that “trying to attribute a residual to a specific factor is an inherently frustrating exercise”, but nevertheless conjectured that “the bulk of the residual increase was attributable to technological change” (p.11).

Following this approach and applying it to the time period 1960–2007, Smith, Newhouse, and Freeland [20] attributed 27–48% of HCE growth in the U.S. to spending on new technologies. Similarly, Di Matteo [8] used regional panel data for the U.S. and Canada for 1975–2000 and regressed real per-capita HCE on income, age structure of the population, and year fixed effects, and found that more than 60% of the growth in HCE could be accounted for by the latter variables. For Germany, Breyer and Ulrich [3] regressed total per-capita HCE of German sickness funds over the period 1970–1995 on GDP, share of population over 65 and time, and found that the latter variable accounted for a 1 percent annual growth rate in HCE, holding everything else constant. Similarly, Breyer et al. [2] regressed real per-capita HCE by age group in Germany over a 12-year period on age, the mortality rate, the (predicted) 5-year survival rate, and time, and found that year fixed effects could be translated into a 2% annual growth rate of HCE. However, since GDP was not in the equation, the mentioned effect also picked up the impact of GDP growth.

The most frequently cited paper in the literature using the *affirmative approach*, the discussion paper by Cutler and McClellan [7], started from the observation that average Medicare reimbursement per heart attack patient in the U.S. rose in real terms by 4% annually between 1984 and 1991 and was thus in line with total per-capita HCE growth in that country, which stood at 4.7%. The authors then showed that the “expansion of intensive cardiac surgeries accounted for essentially all of the growth in treatment costs. In contrast, the real price of heart attack treatments has been nearly constant.” (p.29).

The study most similar to ours is Thorpe and Howard [22]. The authors examined Medicare expenditures over the period 1987–2002 and first observed that two-thirds of the total change in Medicare spending was accounted for by the treatment of 10 common conditions. For each of these conditions, they broke up the total change in expenditures to a change in prevalence and a change in the cost by case.^4^ Interestingly, for some conditions such as cancer, hyperlipidemia, and cerebrovascular disease, almost all the expenditure growth could be attributed to an increase in prevalence, whereas for others (most notably heart disease, trauma and hypertension), the by far predominant factor was a change in cost per case, which reflects technological change.

Our paper also relates to the seminal work by Chandra and Skinner [4], who distinguish three types of medical innovation with respect to their costs and their effectiveness: from inexpensive and highly cost-effective all the way to costly and wasteful. But instead of focusing on innovations, which can be ambiguous in their effects depending upon where they are applied, we look at illness types and examine just the expenditure side, whereas the question of cost effectiveness is beyond the scope of our analysis.

## Research design

The Austrian Bismarck-type health care system guarantees universal access to services for the whole population. With very few exceptions,^5^ the mandatory health insurance covers all expenses for medical care in the outpatient sector, inpatient hospital treatment, and medical drugs. Nine provincial health insurance funds (“Gebietskrankenkassen”) cover insurance for all private-sector employees, retirees, unemployed individuals, and their co-insured dependents. Affiliation with the insurance institution is determined by place of occupation (residence) and, therefore, cannot be freely chosen.

### Data

For our quantitative analysis, we use administrative register data provided by the *Upper Austrian Regional Health Insurance Fund*.^6^ The data include detailed individual inpatient sector information such as the number and length of hospital stays, hospitalization expenditures,^7^ and the patient’s admission diagnosis according to the ICD-10 (International Statistical Classification of Diseases and Related Health Problems) classification system. The data also include individual expenses for medical attendance and medication in the outpatient sector.

The empirical analysis covers the time period from 2005 to 2018. Furthermore, we distinguish survivors from decedents. For survivors, who have at least 4 more years to live, we aggregate the health care expenditures for each calendar year. For decedents, we analyze the last 4 years of life. The final year of life comprises the quarter of death and the three preceding quarters, the second year before death comprises the quarters 4 to 7 before death, and so on. We observe information on the state of insurance for the first day of each quarter and include only observations where the individual is insured in all four quarters, i.e., we exclude individuals with long insurance gaps.

### Empirical strategy

As was mentioned above, we conduct our analysis separately for decedents and survivors. There is no consensus in the literature as to how many years before death the end of life begins. Several authors (e.g, [21]) count the last 2 years towards this period of life, and Zweifel et al. [24] use 3.5 years. To be as general as possible, we alternatively look at HCE in the last 12 months and in the last 48 months of life when we analyze decedents. Our survivor category consists of individuals who have lived at least 4 more years after the particular expenditures incurred. Given that our expenditure data span the years 2005–2018 and the mortality data reach until December 31, 2019, we analyze the following subsamples:

- *Decedents (last year)*: This group includes 116,112 individuals who we observe in the last 12 months of their lives and who died in the period 2005 (last quarter) to 2018.
- *Decedents (last 4 years)*: This group includes 86,870 individuals who we observe during the last 48 months of their lives and who died in the period 2008 (last quarter) to 2018.
- *Survivors*: This group includes 1.416 million individuals who have lived at least 4 more years. As a consequence, we observe their expenditure data between 2005 and 2015 (10.585 million person-years).

Each decedent is linked with a specific diagnosis if the person was treated in a hospital within the period of observation and the diagnosis was the principal diagnosis of this hospital stay. In case of several hospital episodes, we use the last stay before death.^8^ We group the diagnoses by 3-digit ICD-10 codes and use the cut-off criterion for inclusion that the respective ICD code must be relevant for at least 900 cases. This criterion is met by 22 ICD-10 codes comprising in total 42,769 cases or 36.9% of the total decedent (last year) sample. We also analyze diagnoses grouped into important ICD-10 chapters. Here, we use a cut-off of 6000 cases, which is met by 5 ICD-10 chapters.

For the survivor analysis, 1.755 million person-years (16.6%) contain a hospital episode and can therefore be linked to an ICD-10 chapter. Of these, we include only ICD-10 chapters with more than 50,000 cases each (which corresponds to about 0.5% of all person-years). Altogether, these 12 ICD-10 chapters are relevant for 1.56 million cases or 88.8% of all person-years with a hospital stay.

**Table 1:** Upper Austrian sickness fund: members and total HCE

Year	(1)	(2)	(3)
	HCE in mio. €	Members	HCE/member
2005	1,608	1,137,003	1,414
2006	1,716	1,151,143	1,490
2007	1,881	1,163,921	1,616
2008	2,003	1,174,997	1,705
2009	2,068	1,174,869	1,760
2010	2,148	1,180,826	1,819
2011	2,185	1,187,822	1,840
2012	2,288	1,196,435	1,913
2013	2,383	1,208,174	1,972
2014	2,530	1,217,010	2,079
2015	2,633	1,227,854	2,144
2016	2,867	1,245,869	2,301
2017	2,939	1,255,261	2,342
2018	2,959	1,265,631	2,338
Growth rate	0.0460	0.0077	0.0383

This table shows members and expenditures in the Upper Austrian Sickness Fund per year. The bottom line displays the corresponding average annual growth rate

A disease contributes more than proportionally to per-capita HCE whenever

1. its prevalence increases, and/or
2. its cost per case increases faster than overall HCE.

As a prevalence measure, we use the number of cases adjusted by the membership in the health insurance fund. As can be seen from Table 1, membership in the Upper Austrian Health Insurance Fund (OOEGKK) increased from 1.14 million insured persons in 2005 to 1.27 million in 2018, which corresponds to an annual growth rate of 0.77% per year. We subtract this figure from the growth rate in absolute prevalence to determine the relative growth in prevalence. As a benchmark for an over-proportional growth in costs per case, we use the growth rate of expenditures per capita of the sickness fund between 2005 and 2018. The annual growth rate of total expenditures amounts to 4.6%, but since membership grew by 0.77% annually, the per-capita HCE growth rate is 3.83%.^9^

**Table 2:** Distribution of total HCE per year between decedents and survivors

	2006	2007	2008	2009	2010	2011	2012	2013	2014	2015
Decedents (in %)										
Last year of life	9.13﻿	8.92	9.04	9.41	9.47	9.46	9.22	8.66	8.34	8.55
Second year before death	4.68	4.59	4.48	4.71	4.88	4.92	4.70	4.22	4.54	4.36
Third year before death	3.76	3.57	3.74	3.74	3.83	3.94	3.43	3.58	3.32	3.44
Fourth year before death	3.21	3.20	3.12	3.35	3.46	3.11	3.21	2.99	3.05	2.94
Survivors (in %)	79.22	79.72	79.63	78.80	78.36	78.56	79.43	80.56	80.75	80.72

This table shows the distribution of total HCE between survivors and decedents (in percent)

The development of costs per case for a particular disease is calculated as follows: for decedents, we allocate each person to the quarter of his/her death, and for the so-defined group of patients, we determine the average costs in the last 12 (48) months, which we attribute to the respective quarter, which gives

- a time series of length 53 (last quarter of 2005 until last quarter of 2018) for the variable *average HCE in the last year of life*,
- a time series of length 41 (last quarter of 2008 until last quarter of 2018) for the variable *average HCE in the last 4 years of life*.

*Per-capita expenditures by disease group:* To obtain the growth rates per quarter for total HCE, we estimate the following equation:

1 $$\begin{aligned} {\text {ln}}(\bar{h}_t) = \alpha + \beta q + \epsilon _{t} \end{aligned}$$

with $\bar{h}_t$ representing total HCE expenditures in a certain diagnosis-related group (DRG) for people who died in quarter *q*. These expenditures refer to either the decedents’ last or the last 4 years. Total HCE include expenditure for inpatient treatment, medical attendance in the outpatient sector, medication, medical aids, and transport services. The right-hand-side variable *q* represents a linear time trend. The coefficient $\beta$ in the semi-log specification gives the growth rate in HCE per quarter. The annual growth rate is simply the quarterly growth rate multiplied by 4.

**Table 3:** Decedents: average HCE growth rates—last year of life

(1)	(2)	(3)	(4)	(5)	(6)	(7)	(8)	(9)
ICD code	Disease group	Cases	GR cases	Expend. per case	Expend. share (%)	GR cases adjusted	GR expend. per case	GR combined
2	Neoplasms	20,309	1.38	36,646	31.09	0.61	5.44	6.05
19	Injury, poisoning and other external causes	6,462	2.84	20,295	5.49	2.07	3.51	5.59
10	Diseases of the respiratory system	13,314	2.09	22,409	12.38	1.32	3.57	4.89
11	Diseases of the digestive system	6,240	− 0.41	26,412	6.79	− 1.18	3.20	2.02
9	Diseases of the circulatory system	23,762	− 1.25	20,667	20.15	− 2.02	3.55	1.54
N17	Acute renal failure	1,146	6.04	23,645	1.16	5.27	2.74	8.02
J69	Pneumonitis due to solids and liquids	1,458	7.07	22,274	1.36	6.30	1.56	7.87
C50	Malignant neoplasm of breast	1,034	2.60	36,066	1.58	1.83	5.70	7.53
C22	Malignant neoplasm of liver and intrahepatic bile ducts	945	2.75	28,040	1.11	1.98	5.45	7.44
C34	Malignant neoplasm of bronchus and lung	3,435	1.98	34,825	5.02	1.21	6.13	7.35
C25	Malignant neoplasm of pancreas	1,467	2.47	34,668	2.14	1.70	5.46	7.16
I63	Cerebral infarction	2,833	2.93	19,616	2.33	2.16	4.16	6.32
N39	Other disorders of urinary system	1,277	5.45	15,489	0.83	4.68	1.42	6.09
J44	Other chronic obstructive pulmonary disease	1,327	1.44	25,421	1.41	0.67	5.38	6.05
S06	Intracranial injury	1,149	2.24	21,257	1.02	1.47	3.80	5.28
C18	Malignant neoplasm of colon	973	− 0.02	37,739	1.53	− 0.79	5.78	5.00
S72	Fracture of femur	1,831	2.88	20,615	1.58	2.11	2.52	4.63
J18	Pneumonia, organism unspecified	6,002	0.51	20,451	5.04	− 0.26	3.53	3.27
I61	Intracerebral haemorrhage	1,420	0.20	21,714	1.28	− 0.57	3.16	2.59
C78	Secondary malignant neoplasm of respiratory and digestive organs	1,243	− 1.78	32,934	1.68	− 2.55	4.56	2.01
A41	Other sepsis	2,114	− 0.67	34,199	2.98	− 1.44	2.93	1.49
C79	Secondary malignant neoplasm of other and unspecified sites	1,037	− 2.26	38,407	1.61	− 3.03	4.49	1.46
I25	Chronic ischaemic heart disease	1,060	− 1.89	24,632	1.09	− 2.66	3.86	1.20
I50	Heart failure	6,198	− 1.26	19,711	5.01	− 2.03	2.79	0.76
J15	Bacterial pneumonia, not elsewhere classified	1,380	− 3.35	22,418	1.22	− 4.12	4.16	0.04
I21	Acute myocardial infarction	2,410	− 6.49	17,066	1.65	− 7.26	2.57	− 4.69
I26	Pulmonary embolism	1,057	− 7.05	18,390	0.79	− 7.82	2.24	− 5.58

This table summarizes the development of cases and expenditures for disease groups in the last year of life. Column 1 shows the ICD-10 chapter or 3-digit code, column 2 the name of the disease group, column 3 the absolute number of cases, column 4 the annual growth rate of cases, column 5 the average HCE in the last year of life, column 6 the share of total HCE that can be attributed to the disease group, column 7 the adjusted growth rate of cases calculated as column 4 minus 0.77, column 8 the growth rate of expenditures per case ($\beta$ in equation 1 multiplied by 4), and column 9 the combined growth rate calculated as the sum of columns 7 and 8

For survivors with a particular disease (defined by the last hospitalization in the respective year), we attribute total annual HCE to this disease and calculate the average over all patients in this disease group for each year. The annual growth rates for each DRG group can then be calculated analogous to Eq. 1.

## Results

Table 2 shows the distribution of total HCE between survivors and decedents over time in the estimation sample. Approximately 80% of the aggregate expenditures can be attributed to survivors, whereas 9% are spent for patients in their last year of life. The proportion of total HCE caused by patients in their second, third, and fourth year before death is approximately 4.6%, 3.6%, and 3.2%, respectively. The percentages are stable over time.

**Table 4:** Survivors: average HCE growth rates

(1)	(2)	(3)	(4)	(5)	(6)	(7)	(8)	(9)
ICD code	Disease group	Cases	GR cases	Expend. per case	Expend. share (%)	GR cases adjusted	GR expend. per case	GR combined
15	Pregnancy, childbirth and the puerperium	86,457	5.92	4,266	2.37	5.15	4.49	9.64
5	Mental and behavioral disorders	80,803	4.43	12,872	6.66	3.66	5.67	9.33
2	Neoplasms	92,982	2.37	10,193	6.00	1.60	5.75	7.36
13	Diseases of the musculoskeletal system and connective tissue	221,915	2.02	7,450	10.44	1.25	4.84	6.09
18	Symptoms, signs and abnormal clinical and laboratory findings	95,585	1.45	4,367	2.63	0.68	5.12	5.80
7	Diseases of the eye and adnexa	106,376	3.26	4,797	3.22	2.49	3.06	5.55
6	Diseases of the nervous system	83,807	2.05	6,987	3.69	1.28	3.97	5.24
19	Injury, poisoning and other external causes	209,079	0.31	6,206	8.11	− 0.46	5.23	4.77
10	Diseases of the respiratory system	104,208	− 0.71	4,909	3.18	− 1.48	5.47	3.99
9	Diseases of the circulatory system	173,701	0.52	9,558	10.38	− 0.25	4.08	3.83
11	Diseases of the digestive system	178,120	0.04	5,333	5.93	− 0.73	3.93	3.20
14	Diseases of the genitourinary system	124,960	0.08	5,036	3.93	− 0.69	3.71	3.02

This table summarizes the development of cases and expenditures for disease groups of survivors. Column 1 shows the ICD-10 chapter, column 2 the name of the disease group, column 3 the absolute number of cases, column 4 the annual growth rate of cases, column 5 the average annual HCE, column 6 the share of total HCE that can be attributed to the disease group, column 7 the adjusted growth rate of cases calculated as column 4 minus 0.77, column 8 the growth rate of expenditures per case ($\beta$ in equation 1 multiplied by 4), and column 9 the combined growth rate calculated as the sum of columns 7 and 8

### Disease-specific expenditures

The main results for the group of decedents are summarized in Tables 3 and A.2. For decedents in their last year of life [Table 3, column (5)], treatment per case of cancer was most expensive (36,646€), followed by treatment of diseases of the digestive (26,412€) and respiratory system (22,409€). Expenditure growth was over-proportional for neoplasms, diseases of the respiratory system, and injury and poisoning in the sense that treatment of these diseases clearly exceeded the 3.8% overall expenditure growth per sickness fund member and year [see column (9)]. The growth in expenditures for treatment of diseases of the circulatory and digestive system remained significantly behind this overall growth rate.

**Table 5:** Expenditure growth rates (GR) and increase in 5-year survival rates in 11 types of cancer

	Last year		Last 4 years		5-year survival rates (percent)				
	GR expenditures	GR per case	GR expenditures	GR per case	Patients	2000–04	2010–14	Change	
All neoplasms	6.05	5.44	5.59	5.24					
Group 1: strong expenditure growth									
Lung	7.35	6.13	5.99	5.24	56,130	15.4	19.7	4.3	
Pancreas	7.16	5.46	7.92	6.55	18,371	6.7	10.5	3.8	
Breast	7.53	5.70	9.16	5.52	74,818	81.7	84.8	3.1	
Liver and intrahepatic bile ducts	7.44	5.45	8.84	5.29	10,570	11.2	14.8	3.6	
Average (unweighted, weighted)								3.7	3.6
Group 2: other frequent neoplasms									
Skin					19,150	83.4	87.8	4.4	
Leukaemia					31,583	57.6	63.3	5.7	
Ovaries					11,567	40.9	41.0	0.1	
Stomach					19,308	30.0	35.4	5.4	
Colon					46,127	60.7	63.7	3.0	
Rectum					23,360	60.2	64.2	4.0	
Prostate					75,082	90.1	90.2	0.1	
Average (unweighted, weighted)								3.2	2.7

This table compares the growth rates in the 5-year survival rates for different cancer types as provided by CONCORD Working Group and others [6]. We distinguish cancer types with strong expenditure growth (Group 1) from other frequent cancers (Group 2). The growth rates (GR) of expenditures in columns 2–5 are replicated from Tables 3 and A.2

In 9 of the 22 most frequent 3-digit ICD codes, expenditure growth has been at least 2 percentage points higher than the overall growth in expenditures per sickness fund member. These 9 ICD codes contain 4 referring to malignant neoplasms (bronchus and lung, pancreas, liver, and breast) with altogether 6,881 patients (or 6% of all decedents), and in all these cases, the “excessive” expenditure growth derives primarily from a more-than-average growth of costs per case. However, the number of cases is also increased by approximately 2% or more per year.

The non-cancer ICD codes with an over-proportional expenditure growth were chronic obstructive pulmonary disease, pneumonitis, disorders of the urinary system, and acute renal failure but only in the first of these groups expenditures per case grew slightly faster than overall HCE per sickness fund member, whereas in the other three groups, the expenditure growth was exclusively due to an excessive growth of the frequency of cases. We find a substantial expenditure growth for cerebral infarction also (I63). However, the result is accompanied by a strong decline in the number of cases of the neighboring code I64 (stroke, not specified as haemorrhage or infarction) which is not shown in Table 3 due to the lower number of cases (below 900). Taken together, the findings likely reflect a change in coding practices in favor of the more specific I63 at the expense of I64.

The pattern of growth rates in cases and expenditures per case for decedents in their last 4 years of life is very similar, as can be seen in Table A.2 in the Web Appendix. It is remarkable, however, that the expenditure growth rate for pneumonitis runs up to almost 9%. This figure is mainly driven by a more than 8% annual growth in the number of cases.

Turning to the group of survivors, the results in Table 4 reveal that two ICD chapters stand out: conditions related to pregnancy, childbirth, and the puerperium (chapter 15) and treatment of mental and behavioral disorders (chapter 5) showed strong increases in both the number of cases and expenditures per case. The latter result is particularly striking, given that these patients belong to the most expensive ones with average annual expenditures of 12,872€. Finally, neoplasms (chapter 2) show above-average growth of expenditures per patient, which confirms the result for decedents that medical innovation for this group of diseases has been particularly strong over the last 10 years.

The empirical results for a combined analysis of decedents and survivors are basically identical to those for the decedents. We find over-proportional expenditure growth for neoplasms, injury and poisoning, and diseases of the respiratory system. The dynamic development of expenditures for cancer treatment over time further supports the previous findings for the survivors. The results are included in Table A.3 in the Web Appendix.

### The role of expected success

Apparently, expenditure growth for decedents was particularly concentrated at some types of cancer including lung, pancreas, breast, and liver cancer. One possible explanation for the fact that these types of cancer have attracted an increasing share of total resources may be that in these diseases, therapeutic success has improved more than in other diseases, in particular other types of cancer. For cancer types, it is easier to answer this question than for other diseases, because therapeutic success is usually measured using 5-year survival rates (5YSR), and these rates are regularly collected for individual countries and the world as a whole.

CONCORD Working Group and others [6] present data on survival rates for 17 different types of cancer in three time periods (2000–04, 2005–09, and 2010–14) for Austria (and many other countries).^10^ Together with the information on the number of patients diagnosed with any of the neoplasm categories in 2000–2014, we are able to compare the 5-year survival rates (5YSRs) of cancer types with above-average expenditure growth with other frequent cancer types with at least 10,000 patients in this period (see Table 5).

The upper panel of the table depicts that in the group of neoplasms with strong expenditure growth, 5YSRs have all increased about the same, namely between 3.1 and 4.3 percentage points in the 10-year period considered, with a patient-weighted average of 3.6 percentage points. In contrast, the group with less rapid expenditure growth contains cancer types with a strong increase in the 5YSR (such as leukaemia and stomach cancer) and also those without any noticeable increase in the 5YSR (ovaries and prostate cancer). On average, the 2.7 percentage point increase of the patient-weighted 5YSR in the latter group of cancers is a quarter lower than that of the former group (3.6 percentage points). We interpret this difference at least as weak evidence that the expected success may have been one of the factors explaining the rapid expenditure growth in some diseases.

### Age profiles

**Fig. 1:**
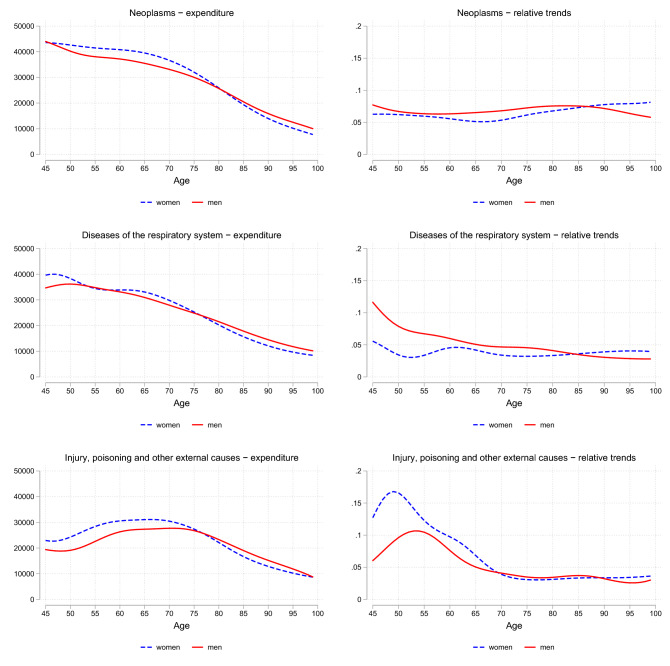
Total HCE for ICD chapters: age profiles and growth rates for decedents. The figures show total HCE per year in € (left panels) and relative changes in total HCE per year in % (right panels) for different ICD chapters by age group for decedents in their last year before death

Finally, we present age profiles for HCE categories with more-than-proportional growth rates. This empirical analysis is based on kernel-weighted local linear regressions for specific age groups and follows Lorenz et al. [18]. All methodological details are included in Web Appendix A.3. Decedents’ per capita expenditures and their growth rates are depicted for neoplasms, diseases of the respiratory system, and for injury and poisoning in Fig. 1. Per-capita expenditures for treatment of cancer and diseases of the respiratory system are highest for the relatively young age group 50–60 in both sexes, whereas the costs for treatment of injury and poisoning peak at ages between 60 and 70 years.

**Fig. 2:**
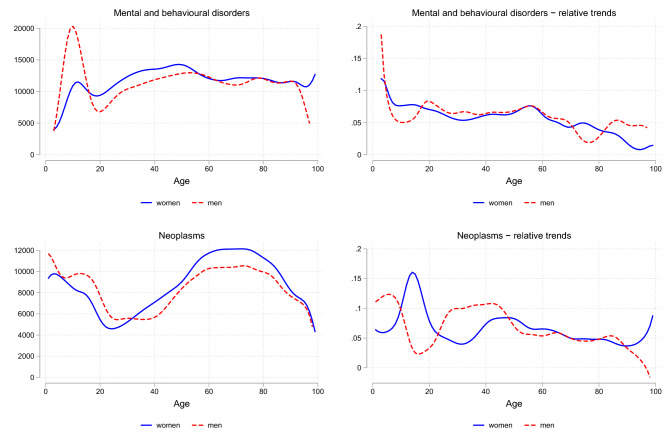
Total HCE for ICD chapters: age profiles and growth rates for survivors. The figures show total HCE per year in € (left panels) and relative changes in total HCE per year in % (right panels) for different ICD-10 chapters by age group for survivors

Our previous results revealed a strong increase in expenditures for cancer treatment over the last years. This increase is not due to a particular age group. Rather, the expenditure growth rates are very similar across ages, which means that the comparatively higher expenditures in the younger age cohorts also grow at the same rate over time. This pattern is somewhat different for treatment of diseases of the respiratory system, in particular for men. The youngest age groups exhibit both the highest per-capita figures and the highest growth rates over time ($\beta _2$ in equation A.2 and shown in the right panels). Expenditures for the treatment of injury, poisoning, and other external causes also reveal stronger growth rates for decedents below 65 years than for older decedents.

With the exception of pregnancy and childbirth costs, we identified the highest expenditure growth in survivors for treatment of mental and behavioral disorders and of neoplasms. Figure 2 reveals that expenditures for mental and behavioral disorders start increasing in very early ages and remain constantly high with levels of more than 10,000€ for all age groups beyond 20. While expenditures increase for all age groups, growth is particularly large for people below 60 with figures of 6–7% per year.

Expenditures for treatment of cancer start to increase at the age 30 for women and 40 for men. The maximum arises in age groups 60–75, indicating that the peaks for survivors occur in older ages than for decedents from cancer. Here, the expenditure growth is particularly pronounced for women between 40 and 50 and for men between 30 and 45 years of age, where they amount to 10% per year and more.^11^

## Discussion, limitations, and conclusions

The present study has analyzed a unique set of high-quality individual data on health care utilization, diagnoses, and time of death of a large number of members of a large regional sickness fund in Austria. In terms of population health and the development of health care expenditures, Upper Austria is basically representative for the whole country, and—to a certain degree—also in a broader European context. Web Appendix A.4 includes statistical material to substantiate this point. Nevertheless, there are a few limitations.

### Limitations

In our analysis, we look at the (dynamics of) expenditures of an average patient suffering from a certain illness. This patient may look different early in the study’s time period than at the end. As an example, if the death rate goes down and the patients that would have died in previous years and now survive are either high spenders or lower spenders, then the mean spending for patients that have died will change. We acknowledge that for certain illnesses, the treated cases have changed considerably and, therefore, the interpretation towards “medical progress or innovation” must be made with caution.

Another topic relates to our process of attributing all health care expenditures to the most recent diagnosis. Obviously, not all expenditures of patients can be assigned to this diagnosis. However, for a considerable share of patients, several health problems can be linked to the same cause, such as cancer spreading to different organs, or heart failure that is preceded by a heart attack. To explore this issue, we examined all hospital stays in decedents’ last year of life. On average, 61.6% of decedents’ hospital days in the last year of life have the same 3-digit ICD-10 diagnosis as our defined end-of-life diagnosis. Furthermore, 70.1% of hospital days are within the same ICD-10 chapter, indicating that health conditions are connected. An exact assignment of different hospitalizations to one cause would require additional information, and most likely a case-by-case assessment by medical professionals.

Although we use high-quality individual-level health register data, the Upper Austrian Health Insurance Fund covers private-sector employees, retirees from the private sector, recipients of unemployment and social security benefits, and their dependents only. The insured persons represent approximately 75% of the whole population, and we do not have information on health care utilization of civil servants, self-employed persons, and farmers.

### Discussion

The main goal of our study was to find out in what disease groups the prevalence of cases and expenditures per case increased in an above-average speed in the time period 2005–2018, and we distinguished between decedents (in their last 4 years of life) and survivors.

The following are the most important findings:

- Among decedents, we observe a disproportionate expenditure growth, which is predominantly driven by a strong increase in costs per case in four types of malignant neoplasms (lung, pancreas, breast, and liver cancer). This is in contrast to the findings of Thorpe and Howard [22] for the time span 1987–2002 and for Medicare patients, according to which the strongest increase in cost per case occurred for patients with hypertension, trauma, arthritis, and heart disease. A possible explanation for the difference is that the study of Thorpe and Howard [22] only encompassed elderly patients and an earlier time period, whereas cancer affects also younger patients and the speed of innovation in cancer therapy has increased in recent years.
- Other diseases with above-average expenditure growth for decedents were COPD, acute renal failure, pneumonitis, and other disorders of the urinary system.
- Among survivors, pregnancies and mental and behavioral disorders showed the most rapid expenditure increase, which stems from a growth in the number of cases and expenditures per case.

To interpret these findings, two important questions have to be answered:

1. Is medical innovation really causal for these developments in health care expenditures?
2. Is it justified to interpret the underlying medical innovations as “medical progress”?

To answer the first question, it has to be shown that no other driving forces have been responsible for the respective expenditure growth. Possible candidates are real income growth, as health care is usually found to be a luxury good (Getzen [12]) and “Baumol’s cost disease”, i.e., the fact that health care provision is labor-intensive and productivity growth low (Baumol [1]). However, both of these forces can serve to explain only the *average* growth differential between health care expenditures and GDP, while we look at disease types for which expenditures have increased significantly faster than general health expenditures. In particular, Baumol’s cost disease applies only to particularly labor-intensive parts of health care provision, which is true in our case only for the treatment of mental and behavioral disorders, which is one of two disease types with above-average expenditure growth for “survivors”. In contrast, the treatment of cancer relies much more heavily on new pharmaceuticals, and the development of personalized medicine has increased the effort exerted for individual patients [11].

Smith et al. [20] analyze the generosity of insurance coverage and its interaction with income and medical technology as another potential driver of health care spending. During our analysis period, there were no changes in the proportion of the population insured, the depth of coverage in insurance contracts or the structure of reimbursement of the health insurance fund to service providers. The mandatory health insurer covers all health care expenditures, and the minor deductibles per hospital stay and for medication have remained constant over time, adjusted for inflation. For this reason, we exclude a significant effect of insurance coverage on expenditure growth for certain types of treatment.

With regard to the second question, it has to be noted that essential contributions of the literature equate medical innovation with “medical progress”. It can be questioned whether this interpretation is valid especially for expenditures in the last year(s) of life. Is not every medical expenditure in the last year of life “wasted” in the sense that it has not succeeded in averting death? And if these expenditures have grown, is it really justified to speak of “medical progress”?^12^ While this objection must be taken seriously, the answer in the case of cancer treatment is not so straightforward. Especially with terminal cancer, a realistic therapeutic goal is not averting, but postponing death, often only by months.^13^ Therefore, part of the expenditure growth even in the last year of life may have been successful on this account. The question also justifies our choice to look alternatively at the last 4 years of life, for which this objection applies even less. We also found that the cancer types for which expenditure growth was particularly pronounced had somewhat higher increase in 5-year survival rates than other cancers, which is a further sign in favor of the interpretation as “progress”.

### Policy implications

A further question relates to possible policy reactions to the observed and analyzed expenditure trends. On one hand, HCE growth that exceeds GDP growth may lead to problems of financing these expenditures from taxes and social insurance contributions. On the other hand, as Hall and Jones (2007) have argued convincingly, higher public expenditures for the treatment of specific diseases may be exactly what citizens want, in particular if these diseases have very special properties (e.g., can occur early in life and cause exceptional anxieties) or have seen spectacular advances in treatment strategies and success in recent times. In the latter case, the appropriate policy reaction could be to accommodate the observed development by speeding up the process of approval of new procedures.

As long as the origin of a disease is predominantly lifestyle-driven, however, the growth of the associated expenditures can be slowed down only in the long run by targeted prevention programs. The same applies to mental and behavioral disorders, which were also found to have caused significant growth in HCE. Yet other groups of diseases such as diseases of the eye and adnexa, of the musculoskeletal and of the genitourinary system might be unavoidable concomitants of aging societies, and the most adequate policy would consist in preparing the public for the necessary increase in tax revenues in the decades to come.

## Supplementary Information

Below is the link to the electronic supplementary material.

Supplementary file1 (PDF 435 KB)

## Footnotes

1 OECD Health Statistics: http://stats.oecd.org/index.aspx?DataSetCode=SHA (10-10-2017)

2 17 years earlier, Harmer [14] wrote a book entitled “The High Cost of Dying”, but this had nothing to do with health care. Instead, the author complained about the costs of funerals.

3 The cause of death specified on the death certificate is often not reliable, in particular if persons die outside a hospital. In Section A.1, we compare the frequencies of end-of-life diagnoses in health registers with the official death register’s causes of death for decedents who we observe in both datasets.

4 They included a third category, change in enrollment, which is not of interest for our study.

5 Patients pay a small daily allowance in hospital and a prescription charge of 5.85€(in 2017) per medical drug.

6 The province of Upper Austria has 1.474 million inhabitants representing 16.7% of the Austrian population. Per capita health care expenditures in 2017 (4012€) were 6.5% below the Austrian average of 4291€ (Hofmarcher and Singhuber [16]). Life expectancy in good and excellent health for women (men) was 6 months higher (lower) than the country mean of 66.7 (65.9) years (Hofmarcher and Molnárová [15]).

7 Hospital expenditures are derived from the Austrian diagnosis-related group (DRG) system. For a few smaller hospitals, we cannot directly observe individual inpatient expenditures. In these (rare) cases, we impute the expenditure information by multiplying a daily allowance fee set by the government to compensate hospitals outside the DRG system with the number of days a patient spent in hospital.

8 Given the relatively short period of data availability and the above-mentioned reliability problems, we do not use the cause of death specified in the Austrian death records. However, for the 28,201 deaths between 2005 and 2010, we can match the end-of-life diagnoses to the official causes of death. As can be seen from Table A.3 in the Web Appendix, the ICD-10 chapter matches the cause of death category in 14,017 cases (49.7%).

9 The growth rates are calculated from regressions of the variables in logs on a linear time trend.

10 Of the cancer types included in Tables 3 and A.2, only ICD code C78 (secondary malignant neoplasms of respiratory and digestive organs) is not listed.

11 The striking peak for women around age 15 is the result of a few expensive cases in the most recent years and should therefore not be taken at face value.

12 We are grateful to the referees for pointing out this problem to us.

13 Krzyszczyk et al. [17] report that in clinical trials, the application of personalized medicine has increased progression-free survival of cancer patients from 2.95 to 5.7 months.

## References

[1] Baumol W (2012). The Cost Disease. Why Computers Get Cheaper and Health Care Doesn’t.

[2] Breyer F, Lorenz N, Niebel T (2015). Health care expenditures and longevity: Is there a Eubie Blake effect?. Eur. J. Health Econ..

[3] Breyer F, Ulrich V (2000). Gesundheitsausgaben, Alter und medizinischer Fortschritt: eine Regressionsanalyse. Jahrbücher für Nationalökonomie und Statistik.

[4] Chandra A, Skinner J (2012). Technology growth and expenditure growth in health care. J. Econ. Lit..

[5] Chernew M, Newhouse J (2012). Health care spending growth. Handb. Health Econ..

[6] CONCORD Working Group and others (2018). Global surveillance of trends in cancer survival 2000–14 (concord-3): analysis of individual records for 37,513,025 patients diagnosed with one of 18 cancers from 322 population-based registries in 71 countries. Lancet.

[7] Cutler, D., McClellan, M.: The determinants of technological change in heart attack treatment. NBER Working Paper No.5751 (1996)

[8] Di Matteo L (2005). The macro determinants of health expenditure in the United States and Canada: assessing the impact of income, age distribution and time. Health Policy.

[9] French E, Kelly E (2016). Medical spending around the developed world. Fiscal Stud..

[10] French E, McCauley J, Aragon M, Bakx P, Chalkley M, Chen SH (2017). End-of-life medical spending in last twelve months of life is lower than previously reported. Health Aff..

[11] Gambardella A (2020). Personalized medicine: recent progress in cancer therapy. Cancers.

[12] Getzen T (2000). Health care is an individual necessity and a national luxury: applying multilevel decision models to the analysis of health care expenditures. J. Health Econ..

[13] Ginzberg E (1980). The high costs of dying. Inquiry.

[14] Harmer R (1963). The High Cost of Dying.

[15] Hofmarcher, M., Molnárová, Z.: Leistungskraft regionaler Gesundheitssysteme. Betrachtung der Bundesländerebene, Health System Intelligence, Research Report (2017)

[16] Hofmarcher, M., Singhuber, C.: Leistungskraft regionaler Gesundheitssysteme. Krankenanstalten im Bundesländervergleich, Health System Intelligence, Fact Book (2019)

[17] Krzyszczyk P (2018). The growing role of precision and personalized medicine for cancer treatment. Technology (Singapur World Science).

[18] Lorenz, N., Ihle, P., Breyer, F.: Aging and health care expenditures: a nonparametric approach (Tech. Rep.). CESifo Working Paper, no. 8300 (2020).

[19] Newhouse JP (1992). Medical care costs: how much welfare loss?. J. Econ. Perspect..

[20] Smith S, Newhouse J, Freeland M (2009). Income, insurance, and technology: why does health spending outpace economic growth?. Health Aff..

[21] Stearns SC, Norton EC (2004). Time to include time to death? The future of health care expenditure predictions. Health Econ..

[22] Thorpe K, Howard DH (2006). The rise in spending among medicare beneficiaries: the role of chronic disease prevalence and changes in treatment intensity. Health Aff..

[23] Zweifel P, Felder S, Meier M (1999). Ageing of population and health care expenditure: a red herring?. Health Econ..

[24] Zweifel P, Felder S, Werblow A (2004). Population ageing and health care expenditure: new evidence on the "red herring.". Geneva Pap. Risk Insur. Issues Pract..

