# Supplementary material for: Looking into the black box of “Medical Innovation”: rising health expenditures by illness type
Source: Eur J Health Econ. 2022 Mar 17;23(9):1601–12. doi: 10.1007/s10198-022-01447-9 (PMC9666302; doi:10.1007/s10198-022-01447-9)
Supplement: Supplementary file 1 — Supplementary file1 (PDF 435 KB) [file 10198_2022_1447_MOESM1_ESM.pdf]

Web Appendix

This Web Appendix provides additional material discussed in the manuscript *Looking into the black box of "Medical Innovation": rising health expenditures by illness type and age* by Friedrich Breyer, Normann Lorenz, Gerald J. Pruckner and Thomas Schober.

A.1 End-of-life diagnosis and official death records

For deaths between 2005 and 2010, we can match the hospital data to the Austrian death records to compare decedents' end-of-life diagnosis with the official cause of death. There are 28,201 deaths where diagnoses from both data sources are available. The ICD-10 3-digit code matches in 6,919 (24.5 %) of the cases, the ICD-10 chapter in 14,017 (49.7 %) of the cases. Table A.1 shows the cross table for diagnoses according to ICD-10 chapters from both data sources.

Table A.1: Comparison of end-of-life diagnosis (EOL) and cause of death

	Cause of death																
EOL	1	2	3	4	5	6	9	10	11	12	13	14	15	17	18	19	Total
1	92	205	5	79	3	28	328	105	114	9	10	42	0	2	8	21	1051
2	8	5641	11	15	4	11	186	41	30	0	2	3	0	0	3	47	6002
3	4	66	12	6	2	4	63	3	7	0	2	2	0	1	3	2	177
4	1	87	1	135	9	17	277	31	27	0	4	11	0	1	15	14	630
5	8	73	1	51	50	46	357	51	30	0	1	10	0	1	15	165	859
6	5	97	0	31	13	157	355	34	17	1	3	2	0	1	6	43	765
7	2	17	1	19	4	3	93	11	3	0	0	1	0	0	2	13	169
8	1	1	0	3	1	0	10	0	1	0	0	0	0	0	0	1	18
9	38	608	5	523	22	96	5509	385	176	5	18	74	1	10	47	135	7652
10	30	564	8	165	32	185	1526	988	92	4	13	45	0	9	36	78	3775
11	44	459	4	62	23	35	537	81	696	1	3	29	0	2	11	51	2038
12	3	39	0	35	5	12	165	21	6	9	1	12	0	0	4	11	323
13	4	48	0	27	5	2	163	29	13	3	22	6	0	0	6	49	377
14	15	207	4	123	6	34	466	55	41	5	7	108	0	0	10	26	1107
15	0	0	0	0	1	0	1	0	1	0	0	0	1	0	0	3	7
16	0	0	0	0	0	0	2	2	0	0	0	0	0	0	0	0	4
17	0	2	0	0	0	0	10	1	1	0	0	0	0	5	0	0	19
18	20	364	1	85	11	47	575	111	67	3	4	25	0	2	18	58	1391
19	9	154	3	64	31	44	737	69	53	3	18	20	0	0	34	574	1813
20	0	0	0	0	0	0	1	0	0	0	0	0	0	0	0	1	2
21	1	10	0	0	1	0	6	2	0	0	0	0	0	0	0	2	22
Total	285	8642	56	1423	223	721	11367	2020	1375	43	108	390	2	34	218	1294	28201

Notes: 1=Certain infectious and parasitic diseases 2=Neoplasms 3=Diseases of the blood and blood-forming organs 4=Endocrine, nutritional and metabolic diseases 5=Mental and behavioural disorders 6=Diseases of the nervous system 7=Diseases of the eye and adnexa 8=Diseases of the ear and mastoid process 9=Diseases of the circulatory system 10=Diseases of the respiratory system 11=Diseases of the digestive system 12=Diseases of the skin and subcutaneous tissue 13=Diseases of the musculoskeletal system and connective tissue 14=Diseases of the genitourinary system 15=Pregnancy, childbirth and the puerperium 16=Certain conditions originating in the perinatal period 17=Congenital malformations, deformations and chromosomal abnormalities 18=Symptoms, signs and abnormal clinical and laboratory findings 19=Injury, poisoning and certain other consequences of external causes 20=External causes of morbidity and mortality 21=Factors influencing health status and contact with health services.

A.2 Additional tables and figures

Table A.2: Decedents: Average HCE growth rates – last four years of life

(1) ICD code	(2) Disease group	(3) Cases	(4) GR cases	(5) Expend. per case	(6) Expend. share (%)	(7) GR cases adjusted	(8) GR expend. per case	(9) GR combined
2	Neoplasms	15,165	1.12	74,572	27.59	0.35	5.24	5.59
19	Injury, poisoning and other external causes	4,952	3.30	43,253	5.23	2.53	2.04	4.57
10	Diseases of the respiratory system	10,298	2.31	52,237	13.05	1.54	2.85	4.40
11	Diseases of the digestive system	4,516	0.31	53,617	5.87	-0.46	2.96	2.49
9	Diseases of the circulatory system	17,543	-1.29	45,774	19.38	-2.06	3.11	1.05
N17	Acute renal failure	934	6.80	51,262	1.18	6.03	3.60	9.63
C50	Malignant neoplasm of breast	751	4.41	87,066	1.64	3.64	5.52	9.16
J69	Pneumonitis due to solids and liquids	1,198	8.33	50,160	1.47	7.56	1.39	8.95
C22	Malignant neoplasm of liver and intrahepatic bile ducts	724	4.33	58,170	1.03	3.56	5.29	8.84
C25	Malignant neoplasm of pancreas	1,137	2.14	63,185	1.75	1.37	6.55	7.92
N39	Other disorders of urinary system	1,051	5.48	41,683	1.07	4.71	1.56	6.27
C34	Malignant neoplasm of bronchus and lung	2,579	1.52	65,770	4.13	0.75	5.24	5.99
S06	Intracranial injury	846	3.55	43,161	0.89	2.78	3.05	5.84
C18	Malignant neoplasm of colon	709	0.15	81,033	1.40	-0.62	6.29	5.67
I63	Cerebral infarction	2,292	0.81	39,766	2.21	0.04	3.81	3.86
S72	Fracture of femur	1,435	3.45	41,943	1.47	2.68	0.90	3.59
J18	Pneumonia, organism unspecified	4,535	1.34	48,904	5.35	0.57	2.57	3.14
I61	Intracerebral haemorrhage	1,057	1.19	42,427	1.09	0.42	2.68	3.10
J44	Other chronic obstructive pulmonary disease	1,026	-0.88	65,523	1.63	-1.65	4.15	2.50
A41	Other sepsis	1,523	0.01	68,011	2.51	-0.76	2.73	1.97
I25	Chronic ischaemic heart disease	761	0.03	52,519	0.98	-0.74	2.59	1.85
I50	Heart failure	4,604	-1.53	47,728	5.28	-2.30	2.74	0.43
C79	Secondary malignant neoplasm of other and unspecified sites	740	-4.28	79,985	1.41	-5.05	4.92	-0.13
C78	Secondary malignant neoplasm of respiratory and digestive organs	927	-5.63	73,883	1.64	-6.40	4.88	-1.51
I21	Acute myocardial infarction	1,593	-5.90	39,715	1.50	-6.67	2.15	-4.52
I26	Pulmonary embolism	684	-6.87	38,890	0.64	-7.64	2.14	-5.51
J15	Bacterial pneumonia, not elsewhere classified	1,027	-9.28	51,093	1.22	-10.05	4.04	-6.01

Notes: This table summarizes the development of cases and expenditures for disease groups in the last four years of life. Column 1 shows the ICD-10 chapter or 3-digit code, column 2 the name of the disease group, column 3 the absolute number of cases, column 4 the annual growth rate of cases, column 5 the average HCE in the last four years of life, column column 6 the share of total HCE that can be attributed to the disease group, column 7 the adjusted growth rate of cases calculated as column 4 minus 0.77, column 8 the growth rate of expenditures per case (β in equation 1 multiplied by 4), and column 9 the combined growth rate calculated as the sum of columns 7 and 8.

Table A.3: Decedents and survivors: Average HCE growth rates

(1) ICD code	(2) Disease group	(3) Cases	(4) GR cases	(5) Expend. per case	(6) Expend. share (%)	(7) GR cases adjusted	(8) GR expend. per case	(9) GR combined
2	Neoplasms	159,584	1.43	13,080	10.39	0.66	5.79	6.44
19	Injury, poisoning and other external causes	230,147	0.42	6,563	7.47	-0.35	5.10	4.75
10	Diseases of the respiratory system	147,560	-0.09	7,078	5.15	-0.86	4.80	3.94
11	Diseases of the digestive system	198,150	-0.09	6,094	5.96	-0.86	3.77	2.90
9	Diseases of the circulatory system	250,707	-0.27	9,987	12.34	-1.04	3.85	2.82

Notes: This table summarizes the combined development of cases and expenditures for survivor and decedent observations (last four years of life, included separately as annual observations) from 2005 to 2015. Column 1 shows the ICD-10 chapter, column 2 the name of the disease group, column 3 the absolute number of cases, column 4 the annual growth rate of cases, column 5 the average HCE in the last year of life, column 6 the share of total HCE that can be attributed to the disease group, column 7 the adjusted growth rate of cases calculated as column 4 minus 0.77, column 8 the growth rate of expenditures per case (β in equation 1 multiplied by 4), and column 9 the combined growth rate calculated as the sum of columns 7 and 8.

A.3 Age, HCE, and HCE growth

To analyze the age profiles of HCE for survivors and decedents, we apply local regression techniques. In particular, we follow Lorenz et al. (2020) and calculate smoothed values of HCE over the lifecycle using kernel-weighted local linear regressions. The idea is to use a weighted average of observations close to a specific age to derive a non-parametric estimate of the according expenditures. We pool the available annual observations and estimate the weighted regression

$$h_{it} = \beta_0 + \beta_1(a_0 - a_i) + \beta_2 t + \epsilon_{it}, \tag{A.2}$$

for the expenditures h of individual i at time t separately for the age groups a_0 ranging from 0 to 99, allowing for linear effects of ages in the neighborhood of a_0 $(a_0 - a_i)$ and a time trend t. Consequently, β_0 is a non-parametric estimate of expenditures in age group a_0. We also report β_2/β_0 as an estimate for the age-specific expenditure growth rate per year.[1] Estimations are run for different expenditure components and disease groups separately.

[1] For the estimation weights, we follow Lorenz et al. (2020) and use the normal kernel with a constant bandwidth (i.e., the bandwidth is the same for all age classes). Because of the higher number of observations and a lower variance of the dependent variable, a smaller bandwidth can be employed for survivors than for decedents.

A.4 Life expectancy and health care expenditure in Upper Austria

Eurostat data[2] show that life expectancy at birth in Austria (the solid black line in Figure A.1) lies within the upper range of all EU-28 countries. However, there is a series of countries such as Sweden, Finland, France, Italy, or Spain with significantly higher life expectancy.

Figure A.1: Life expectancy at birth in EU-28 countries

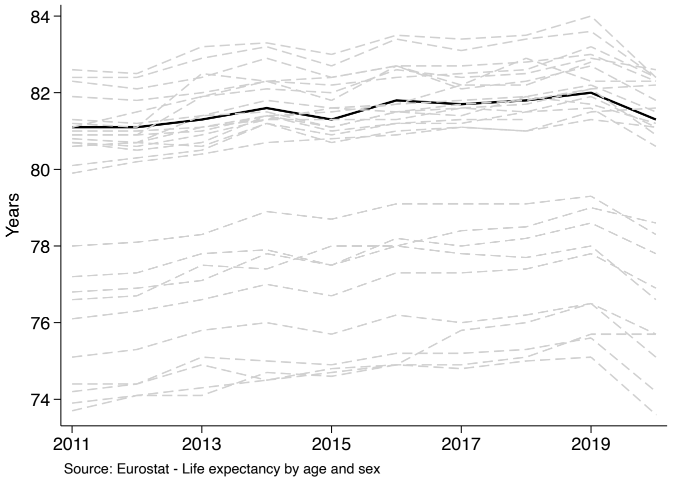

Within the country, Austria shows a clear east-west divide in terms of life expectancy, as can be seen in Table A.4. The highest values are found in the western provinces of Vorarlberg and Tyrol, while life expectancy is lowest in Vienna. Upper Austria has been very close to the Austrian average for many years.

Table A.4: Life expectancy at birth in Austrian provinces (in years)

	Women				Men			
	2005	2010	2015	2020	2005	2010	2015	2020
Austria	82.20	83.13	83.59	83.74	76.61	77.66	78.63	78.94
Burgenland	81.80	83.38	83.77	83.84	76.35	77.55	78.31	79.80
Carinthia	82.66	83.53	83.98	84.23	76.78	77.65	78.35	78.77
Lower Austria	81.71	83.04	83.22	83.61	76.41	77.56	78.52	78.88
**Upper Austria**	**82.32**	**83.45**	**83.76**	**84.08**	**76.89**	**77.97**	**78.91**	**79.26**
Salzburg	82.33	83.84	84.61	84.44	76.83	78.54	79.63	79.89
Styria	82.70	83.49	83.76	83.93	76.60	77.74	78.50	78.58
Tyrol	83.19	84.01	84.33	84.48	77.81	79.00	80.02	80.12
Vorarlberg	83.40	84.12	84.43	84.51	77.90	78.79	79.83	80.18
Vienna	81.54	81.85	82.77	82.72	75.90	76.52	77.63	77.84

Source: Statistics Austria, Population Statistics

Figure A.2 provides information on the level and growth of health care spending in Austria (the solid black line) and EU-28 countries based on the WHO Global Health

[2] `https://appsso.eurostat.ec.europa.eu/nui/show.do?dataset=demo_mlexpec&lang=en`

Expenditure Database (GHED).[3] The figures illustrate that Austria is one of the countries in Europe with the highest per capita health expenditure. At the same time, it can be seen that the growth rates of per capita expenditure over time correspond with EU-28 averages or even lie in the lower range. Figure A.3 displays levels and growth rates of per capita fund-financed hospital spending in the Austrian provinces over the last 10 years.[4] Expenditures and their growth rates in Upper Austria (the solid black line) correspond well with nationwide averages.

Figure A.2: Current health expenditures per capita (CHE)

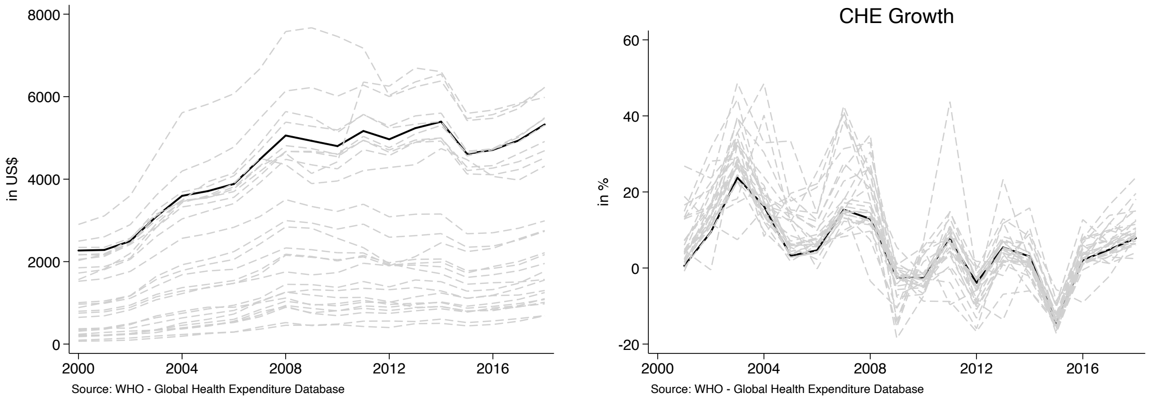

Figure A.3: Fund-financed hospital expenditures per capita (FHE)

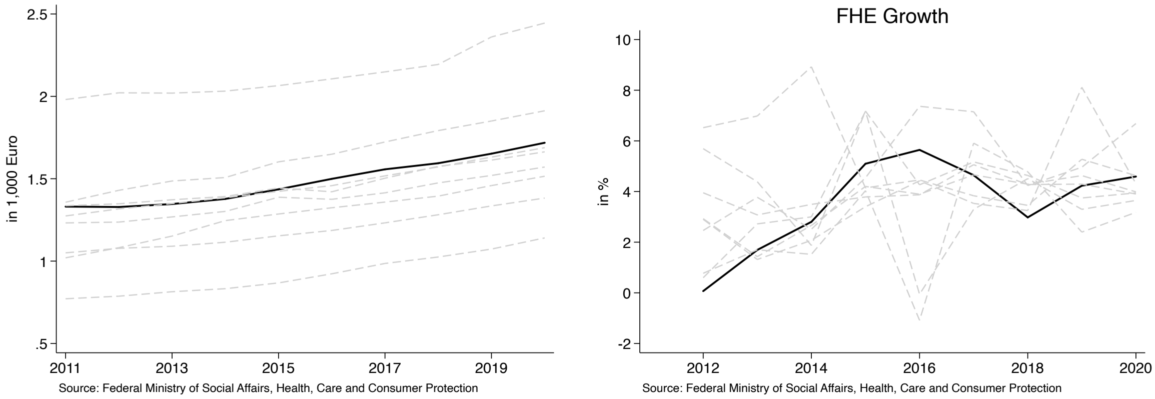

[3] https://apps.who.int/nha/database
[4] http://www.kaz.bmg.gv.at/kosten.html
